# Robust Optimization of SBRT Planning for Patients With Early Stage Non-Small Cell Lung Cancer

**DOI:** 10.1177/1533033820916505

**Published:** 2020-04-21

**Authors:** Haijiao Shang, Yuehu Pu, Yuenan Wang

**Affiliations:** 1 Shanghai Institute of Applied Physics, Chinese Academy of Sciences, Shanghai, People’s Republic of China; 2 University of Chinese Academy of Sciences, Beijing, People’s Republic of China; 3 Department of Radiation Oncology, National Cancer Center/National Clinical Research Center for Cancer/Cancer Hospital, Shenzhen Hospital, Chinese Academy of Medical Sciences and Peking Union Medical College, Shenzhen, People’s Republic of China

**Keywords:** stereotactic body radiotherapy (SBRT), robust optimization (RO), dose volume histogram bands width (DVHBW), radiation pneumonitis (RP)

## Abstract

**Purpose:**

Setup uncertainty is a known challenge for stereotactic body radiotherapy planning. Using the internal target volume-based robust optimization was proposed as a more accurate way than the conventional planning target volume-based optimization when considering the robustness criteria. In this study, we aim to investigate the feasibility of internal target volume-based robust optimization in stereotactic body radiotherapy planning using 4-dimensional computed tomography and develop a novel dose–volume histogram band width metric to quantitatively evaluate robustness.

**Method and Materials:**

A total of 50 patients with early stage non-small cell lung cancer, who underwent stereotactic body radiotherapy, were retrospectively selected. Each of the 50 patients had 2 stereotactic body radiotherapy plans: one with the conventional planning target volume-based optimization and the other with patient-specific robustly optimized internal target volume and with a uniform 5 mm setup error. These were compared with the planning target volume-based optimization method based on both plan quality and robustness. The quality was evaluated using dosimetric parameters and radiobiology parameters, such as high-dose spillage (*V*_90%RX_, conformity index), intermediate-dose spillage (dose falloff products), low-dose spillage (normal tissue: *V*_50%RX_), and lung tissue complication probability. The robustness was evaluated under a uniform 3 to 5 mm setup errors with a novel proposed metric: dose–volume histogram band width.

**Results:**

When compared with planning target volume-based optimization plans, the internal target volume-based robust optimization plans have better conformity of internal target volume coverage (conformity index: 1.17 vs 1.27, *P* < .001), intermediate-dose spillage (dose falloff product: 129 vs 167, *P* < .001), low-dose spillage in normal tissue (*V*_50%RX_: 0.8% vs 1.5%, *P* < .05), and lower risk of radiation pneumonitis (lung tissue complication probability: 4.2% vs 5.5%, *P* < .001). For the robustness, dose–volume histogram band width analysis shows that the average values in internal target volume, *D*_95%_, *D*_98%_, and *D*_99%_, of internal target volume-based robust optimization are smaller than that of planning target volume-based optimization (unit cGy) under 3-, 4-, and 5-mm setup uncertainties (3-mm setup uncertainty: 42 vs 73 cGy; 4-mm setup uncertainty: 88 vs 176 cGy; 5-mm setup uncertainty: 229 vs 490 cGy), which might indicate that internal target volume-based robust optimization harbored a greater robustness regardless of the setup errors.

**Conclusions:**

Internal target volume-based robust optimization may have clinical potential in offering better plan quality in both target and organs at risk and lower risk of radiation pneumonitis. In addition, the proposed internal target volume-based robust optimization may demonstrate robustness regardless of different setup uncertainties in the stereotactic body radiotherapy planning.

**Registration:**

Retrospective study with local ethics committee approval.

## Introduction

Stereotactic body radiation therapy (SBRT) involves the delivery of a single high dose radiation treatment or few fractionated radiation treatments, which showed high local control in tumors.^1,2^ According to the International Commission Radiological Units (ICRU) report, the concept of the planning target volume (PTV) is commonly used to account for tumor motion and setup uncertainty.^3^ The internal target volume (ITV) is technically the motion envelope encompassing the clinical target volume (CTV).^3,4^

However, the optimal margin for the setup uncertainty from ITV to PTV is controversial, as it can be reduced by updating the image-guided radiation therapy (IGRT) equipment. Cristina Garibaldi *et al* ^5^ found that setup uncertainties could be brought down to about 3 mm, in all directions, using a cone-beam computed tomography (CT). Richmond *et al* ^6^ recommended an ITV expansion margin of 4 mm, in all directions, to compensate for residual setup uncertainties in image-guidance and intrafraction motion for patients immobilized in a body-fix device. Therefore, a conventional SBRT plan optimized with a 5-mm uniform margin PTV on the ITV may unnecessarily increase the volume of the irradiated normal tissue (NT). An additional problem is that the PTV concept as typically applied in intensity modulated radiotherapy (IMRT) planning relies on the so-called static dose cloud approximation. The underlying assumption is that the dose distribution in the treatment room coordinates is not affected by changes in the patient’s anatomy. That is, it is assumed that the CTV receives the prescribed dose if it stays within the PTV. This fundamental assumption is not generally fulfilled and is violated.

Various efforts have been made by previous studies to reduce the PTV margin. Li *et al* ^7^ proposed a true 4-dimensional planning method (t4Dplan) on IMRT using 4-dimensional computed tomography (4DCT) data sets to maximize the sparing of critical structures. The rationale of the t4Dplan method is to deliver a smaller dose to the tumor when the tumor is at its T0 position and deliver higher doses when it is close to its T50 position. This will compensate for the under dosing at the T0 position, given that patients spend more time in the T50 phase than in the T0 phase during the entire respiratory cycle. Their studies indicate that the t4Dplan method does not need a target margin, which improves the sparing of normal structures. Suh *et al* ^8^ developed a 4-dimensional (4D)-IMRT treatment planning method by modifying and applying a dynamic multileaf collimator motion-tracking algorithm. A 4D treatment plan was created based on a 4DCT image that was flexible enough to account for changes in patient setup during the treatment. These gating and tracking strategies were effective in reducing the volume of the irradiated target; however, they resulted in markedly extended treatment times and could not be widely used in clinical practice.

Recently, a new strategy to deal with the setup uncertainty, that uses a robust optimization algorithm instead of PTV margin-based optimization, has been reported to be effective in compensating for the setup uncertainty in treatment planning. Zhang *et al* ^9^ evaluated CTV-based robust optimization for lung cancer, and the results demonstrated that a robust optimization can generate plans that offer increased sparing of organ at risk (OAR); especially, for normal lung tissue and OARs near the target. Liang *et al* ^10^ evaluated ITV-based robust optimization (RO-ITV) for lung cancer with SBRT planning and showed that it can account for setup uncertainties.

In our study, we extended the RO-ITV technique to SBRT planning by accounting for plan quality and plan robustness. Innovation is mainly carried out from the following 2 aspects: On one hand, the plan quality considers more details that are of clinical concern, such as dose falloff product (DFP) for dose sharpness and the normal tissue complication probability (NTCP) model for radiation pneumonitis (RP). On the other, we developed a new metric, which is the dose–volume histogram band width (DVHBW), as an independent parameter (or dosimetric endpoint) to quantitatively assess the robustness of the plan, and whether its robustness is affected by updating the IGRT equipment. The proposed DVHBW method provides a simple metric to compare the robustness of different SBRT plans with statistical evaluation.

## Materials and Methods

### Patient and Plan Data Characteristics

A total of 50 patients with early stage (IA/IB) non-small cell lung cancer (NSCLC) were treated with SBRT and retrospectively selected for this study, which was approved by the local institutional research review board. Gross target volumes (GTVs) were contoured by the attending radiation oncologists, on each phase of the 4DCT, and ITVs were created by encompassing the extent of 10 GTV motion in all 10 phases of 4DCT. The ITV was expanded with the setup uncertainty from the PTV by including a setup uncertainty of 5 mm in the superior–inferior, anterior–posterior (AP), and left–right (LR) directions.^11^ The median ITV volume was 19.94 cm^3^ (range of 0.92-70.82 cm^3^) and the median PTV volume was 47.56 cm^3^ (range of 5.87-133.20 cm^3^).

All clinical SBRT plans used the VMAT (RapidArc) technique with the Edge linear accelerator (Linac; Varian Medical Systems, Palo Alto, California) and a 120-leaf multileaf collimation (MLC) flattening filter free mode (6 MV and 1200 MU/min dose rate). The treatment plans were created in the Eclipse TPS version11 (Varian Medical Systems) with the prescription dose of 50 Gy in 4 fractions. A summary of the patients’ information is listed in Table 1. The dose prescription was 50 Gy in 4 fractions, that is, 12.5 Gy per fraction.

**Table 1.** Patient and Peripheral NSCLC Characters (Tumor Size, Prescription).

Patient No.	Tumor Stage	ITV, cm^3^	PTV, cm^3^	Prescription Dose, Gy
1	IA	23.44	47.05	12.5 Gy × 4fx
2	IA	7.77	24.83	12.5 Gy × 4fx
3	IB	34.63	94.12	12.5 Gy × 4fx
4	IA	4.18	17.79	12.5 Gy × 4fx
5	IA	4.26	15.14	12.5 Gy × 4fx
6	IB	27.11	84.12	12.5 Gy × 4fx
7	IB	37.35	85.54	12.5 Gy × 4fx
8	IA	4.81	19.16	12.5 Gy × 4fx
9	IA	7.71	24.76	12.5 Gy × 4fx
10	IB	27.36	48.06	12.5 Gy × 4fx
11	IB	47.55	91.28	12.5 Gy × 4fx
12	IA	0.92	5.87	12.5 Gy × 4fx
13	IB	13.97	38.01	12.5 Gy × 4fx
14	IB	70.82	133.2	12.5 Gy × 4fx
15	IB	36.49	82.26	12.5 Gy × 4fx
16	IB	35.43	71.59	12.5 Gy × 4fx
17	IB	29.23	51.25	12.5 Gy × 4fx
18	IB	33.33	87.26	12.5 Gy × 4fx
19	IA	9.52	28.62	12.5 Gy × 4fx
20	IB	26.66	63.35	12.5 Gy × 4fx
21	IA	19.73	48.06	12.5 Gy × 4fx
22	IB	47.98	90.31	12.5 Gy × 4fx
23	IA	9.04	29.96	12.5 Gy × 4fx
24	IA	3.69	11.33	12.5 Gy × 4fx
25	IB	30.24	52.15	12.5 Gy × 4fx
26	IA	2.95	13.27	12.5 Gy × 4fx
27	IB	64.45	125.02	12.5 Gy × 4fx
28	IA	8.24	24.78	12.5 Gy × 4fx
29	IA	6.89	22.65	12.5 Gy × 4fx
30	IA	17.22	40.32	12.5 Gy × 4fx
31	IA	20.15	53.47	12.5 Gy × 4fx
32	IB	56.43	98.56	12.5 Gy × 4fx
33	IA	8.67	27.69	12.5 Gy × 4fx
34	IB	52.65	93.57	12.5 Gy × 4fx
35	IA	5.88	22.65	12.5 Gy × 4fx
36	IB	62.86	119.68	12.5 Gy × 4fx
37	IA	3.56	10.74	12.5 Gy × 4fx
38	IA	12.62	36.66	12.5 Gy × 4fx
39	IB	36.88	80.98	12.5Gy × 4fx
40	IB	66.86	124.68	12.5 Gy × 4fx
41	IB	49.89	96.68	12.5 Gy × 4fx
42	IA	4.89	20.38	12.5 Gy × 4fx
43	IB	32.62	60.88	12.5 Gy × 4fx
44	IA	3.88	12.28	12.5 Gy × 4fx
45	IB	46.66	88.62	12.5 Gy × 4fx
46	IA	6.84	20.66	12.5 Gy × 4fx
47	IA	5.68	21.28	12.5 Gy × 4fx
48	IA	16.56	42.66	12.5 Gy × 4fx
49	IA	12.42	32.86	12.5 Gy × 4fx
50	IB	58.88	102.68	12.5 Gy × 4fx
Median (range)		19.94 (0.92-70.82)	47.56 (5.87-133.20)	

Abbreviations: ITV, internal target volume; NSCLC, non-small cell lung cancer; PTV, planning target volume.

### Treatment Planning

All patients data (Image sets, RtStructure) are imported into the RayStation (Ver.8B, Raysearch Labs, Sweden), and 2 types of SBRT plans, using PTV-based conventional optimization (CO-PTV) and RO-ITV, are regenerated using the same beam geometry as clinical SBRT plans in Raystation. However, the prescribed dose of all plans is directly delivered to the ITV, with each plan normalized such that 100% of the prescribed dose covered 95% of the ITV. Moreover, patient setup uncertainty is differently handled; while the RO-ITV plan using a robust optimization algorithm is based on ITV. As described by Fredriksson *et al* ^12-14^, the robust optimization in RayStation is a minimax optimization. The minimax optimization method minimizes the objective function value such that the prescription dose is valid even in the worst-case scenario and with respect to setup uncertainties. In practice, over 95% of the ITV volume received prescription dose (*D*_95%_ ≥ 5000 cGy) and 99% of the ITV volume received 90% of prescription dose (*D*_99%_ ≥ 4500 cGy) are used in the robust function, in which patient setup uncertainties are taken into account by shifting the plan isocenter 5 mm in the LR direction, 5 mm in the inferior-superior direction, and 5 mm in the AP direction. Thus, the worst-case robust optimization took into account 6 setup uncertainties and generated SBRT plans by minimizing the penalty of the worst cases for the ITV. Another important point is that, in order to minimize dose spillage, auxiliary structures, such as 2 rings compassing the target area, should have lower dose limits compared with CO-PTV. This is due to the volume of the ITV, which is smaller than that of the PTV, and which results in steep gradient falloffs, away from the target edge.

The interfraction motion is generally more complex to model and is often modeled as a setup error.^15^ A treatment plan, which only considers fixed setup errors, yields a dose distribution which is comparable to a CO-PTV. Actually, setup errors might be averaged over the course of treatment and most studies in the IMRT model the setup errors using Gaussian distributions in 3 dimensions. The integration of uncertainties, with setup errors into the optimization process, induces an automatic expansion of CTV that expect a dose, which covers enough target under uncertainties. Sir *et al* ^16^ investigated in detail the setup errors of what the shape of the dose falloff, at the edge of the target volume, depends on.

### Plan Quality Evaluation

Both RO-ITV and CO-PTV plans were normalized to have 5000 cGy at ITV of *D*_95%_. The dosimetric parameters, including ITV dose coverage, conformity index (CI), homogeneity index (HI), DFP, and OARs are compared for RO-ITV and CO-PTV according to ICRU 0915.

The ITV dose coverage is described as 99% of the target volume (ITV) that received a minimum of 90% of the prescription dose (ITV: *V*_90%RX_ ≥ 99%).

The CI is calculated using Equation 1:

1 $$\text{CI}=\frac{\text{VRI}}{\text{TV}}\;$$

Where VRI is the prescription isodose volume and TV the ITV volume.

The HI is calculated using Equation 2:

2 $$\text{HI}=\frac{D_{2\text{\%}}-D_{98\text{\%}}}{D_{50\text{\%}}}$$

Where *D*_2%_, *D*_98%_, and *D*_50%_ are the doses that cover 2%, 98%, and 50% of the ITV, respectively. The DFP, defined as the product of *R*_50%_ and *D*2cm, was found to have a weak relationship with tumor size and is therefore used to compare the intermediate-dose spillage of the SBRT plan.^17^

DFP is expressed by Equation 3:

3 $$\text{DFP}=R_{50\text{\%}}\times D2\text{cm}$$

Where *R*_50%_ is the ratio of 50% of the prescription isodose volume to the ITV and *D*2cm is the maximum dose at 2 cm from the ITV in any direction.

The OARs are also evaluated using dose–volume histogram (DVH) metrics, such as lung (*D*_mean_, *V*_20_), spinal cord *D*_1cc_ and NT (*V*_50%RX_), where NT is a ring with body minus boundary that is away from ITV is 2 cm; *V*_50%RX_ is NT volume received a minimum prescription dose of 50%.

### Toxicity Assessment

The dose–volume relationships for lung complications has been studied for several years in lung cancer. The popular Lman normal tissue complication (NTCP) model was used to fit the dose–volume relationship to the clinical data. However, there are large uncertainties in the NTCP model and its associated model parameters, which might reflect subjectivity in the scoring complications, disparity in end points, and dosimetric uncertainties. In current studies, the Lyman NTCP model in the Moiseenko studies^18^ is used to assess the dependence of lung complications incidence on the dose–volume for both RO-ITV and CO-PTV plans. The parameters details are presented in Table. 2. In the Moiseenko studies, long survival allowed the assessment of lung complications data in patients with thymoma for acute and late response, which make the model parameters best suited for their data.

**Table 2.** Parameters Used in the Lyman-Kutcher-Burman Model.

Parameter	Volume	D50, cGy	M	N	α/β, Gy
NTCP	Both lungs	2190	0.37	0.8	3

Abbreviation: NTCP, normal tissue complication probability.

### Robustness Quantification

Although a patient setup uncertainty of 5 mm was introduced for both CO-PTV and RO-ITV in SBRT planning, it was necessary to study the situation in which the setup uncertainty is less than 5 mm in the evaluation of robustness, which could be caused by updating the IGRT equipment. Therefore, the evaluation of robustness in SBRT planning started with the creation of 3 scenarios or setup groups whose details are as follows:

1. *3-mm setup group*: nominal scenario (no setup uncertainty) and 14 uncertainty scenarios (6 with 3 mm in X, Y, and Z directions, respectively; and 8 with 3 mm in diagonal directions) that simulate a 3-mm maximum setup isotropic space.
2. *4-mm setup group*: nominal scenario (no setup uncertainty) and 14 uncertainty scenarios (6 with 4 mm in X, Y, and Z directions, respectively; and 8 with 4 mm in diagonal directions) that simulate a 4-mm maximum setup isotropic space.
3. *5-mm setup group:* nominal scenario (no setup uncertainty) and 14 uncertainty scenarios (6 with 5 mm in X, Y, and Z directions, respectively; and 8 with 5 in diagonal directions) that simulate a 5-mm maximum setup isotropic space.

Hence, a total of 15 perturbed dose distributions were computed for each setup group. In the case of the ITV, the metrics included the DVHBW.

The DVHBW can be expressed by Equation 4:

4 $$\Delta D_{x}=\text{max}\left(D_{x}^{s}\right)-\text{min}\left(D_{x}^{s}\right)\;$$

Where $D_{x}^{s}$ is the DVH metric of the ITV in*x* relative volume (%) for setup group  s. In short, the DVHBW represents the variation of DVH metrics in a setup group. In our studies, the values of *x* are 95, 98, and 99, due to the sensitivity of the DVH metric, in the ITV coverage, to the setup uncertainty in the SBRT planning. A total of 3 DVHBWs were obtained in the ITV target (*D*_95_, *D*_98_, *D*_99_) under 1 setup group, in which it was separately compared between the RO-ITV and CO-PTV plans. Furthermore, the mean DVHBW was calculated by averaging 3 DVHBWs for 1 group.

Another metric, the voxel-wise dose distribution (VWDD),^19^ was introduced for plan robustness. The VWDD-min as an aggregate dose distribution based on the minimum dose value, in each voxel and over all scenarios, was used to evaluate the target coverage. The VWDD-max, which is an aggregate dose distribution based on the maximum dose value, in each voxel over all scenarios, was used to evaluate OAR sparing.

### Statistical Analysis

The student *t* test was carried out to compare the pairwise difference between 2 treatment techniques (CO-PTV and RO-ITV) in term of plan quality, risk of RP, and plan robustness. A *P* value < .05 was considered statistically significant. A study was performed using a nonlinear model with Excel software version (v.2016, Microsoft; https://www.microsoft.com) to evaluate the correlation between the mean DVHBW and 3 setup groups.

## Results

### Target Dose Coverage

**Figure 1.**
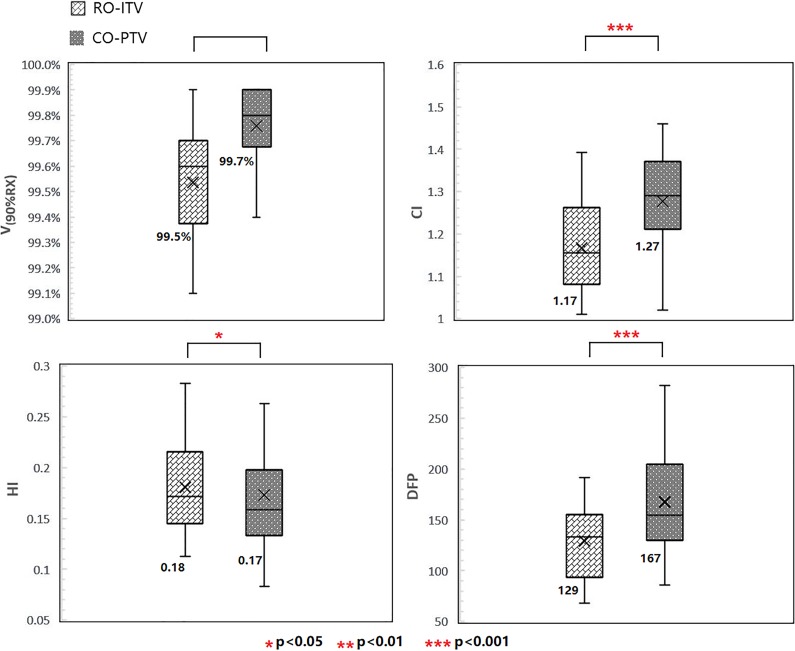
Plan quality comparison between RO-ITV and CO-PTV in *V*_90%RX_, HI, CI, and DFP. CI indicates conformity index; CO-PTV, planning target volume-based conventional optimization; DFP, dose falloff product; HI, homogeneity index; RO-ITV, internal target volume-based robust optimization.

In general, RO-ITV met the ITV coverage and critical organ dose objectives according the Radiation Therapy Oncology Group (RTOG) 0915 were used for the SBRT plans.^11^ In Figure 1, more details are shown, comparing RO-ITV to CO-PTV.

The most important target coverage parameters, *V*_90%RX_ and *V*_100%RX_, are satisfied by both RO-ITV and CO-PTV plans, with no significant differences between them.

1. An acceptable CI was defined as <1.2 and <1.5 for a deviation in RTOG 0915. The value of CI for RO-ITV was 1.17 for RO-ITV versus 1.27 for CO-PTV (*P* < .001; for ITV).
2. Although a HI was not required by RTOG 0915, we observed that the HI was 0.18 for RO-ITV versus 0.17 for CO-PTV (*P* < .05).
3. The falloff gradient beyond the ITV that extends into NT structures should be steep in all directions and meet *R*_50%_ and *D*2cm according to RTOG 0915. The DFP, as an effective metric and regardless of tumor size, was investigated and found to be better in RO-ITV. For ITV, it was 129 for RO-ITV versus 167 for CO-PTV (*P* < .001).

### Organ at Risk and RP

**Figure 2.**
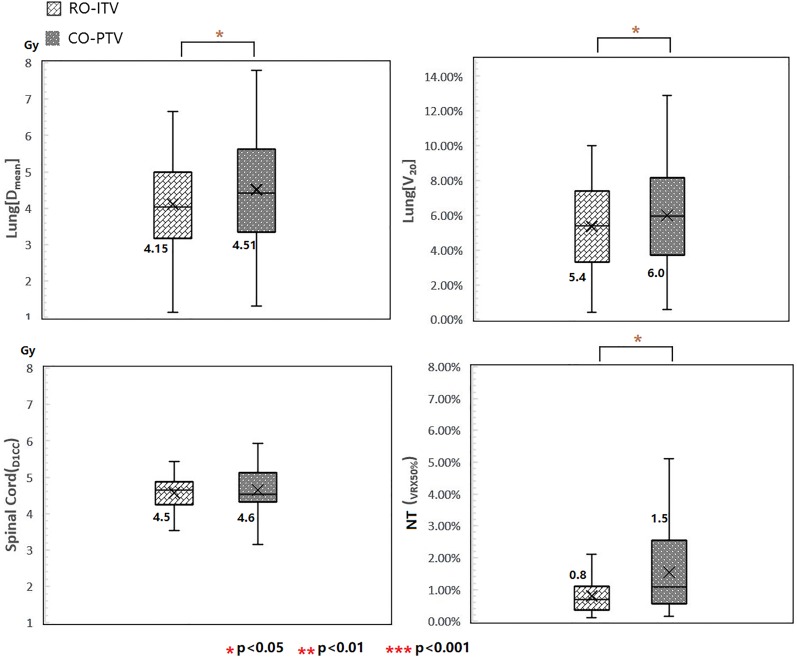
Plan quality comparison between RO-ITV and CO-PTV in lung (*D*_mean_), lung (*V*_20_), spinal cord (*D*_1cc_), and NT (*V*_50%RX_). CO-PTV indicates planning target volume-based conventional optimization; NT, normal tissue; RO-ITV, internal target volume-based robust optimization.

Many dosimetric OAR parameters were lower for RO-ITV compared to CO-PTV (Figure 2). The mean dose in lung tissue (*D*_mean_) was 415 cGy for RO-ITV compared to 451 cGy for CO-PTV (*P* < .05) and the lung *V*_20_ values were 5.4% for RO-ITV compared to 6.0% for CO-PTV (*P* < .05). In addition, the 50% prescription isodose volume (*V*_50%RX_) in the NT was significantly lower when irradiated with the RO-ITV plans (*P* < .05).

**Figure 3.**
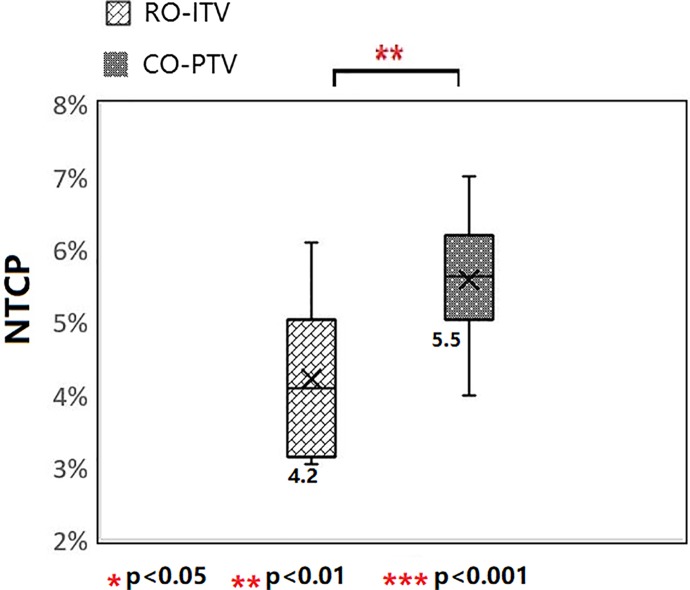
NTCPs for lung in both RO-ITV and CO-PTV (The y-axis is a % probability). CO-PTV indicates planning target volume-based conventional optimization; NTCP, normal tissue complication probability; RO-ITV, internal target volume-based robust optimization.

The SBRT plan with the RO-ITV methods revealed significantly superior mean NTCP values for the lung (NTCP = 4.2%) compared to CO-PTV (NTCP = 5.5%; *P* < .05; Figure 3). Consequently, the RO-ITV demonstrated a lower risk of RP compared to the CO-PTV technique for the lung.

### Robustness Analysis: DVHB

**Figure 4.**
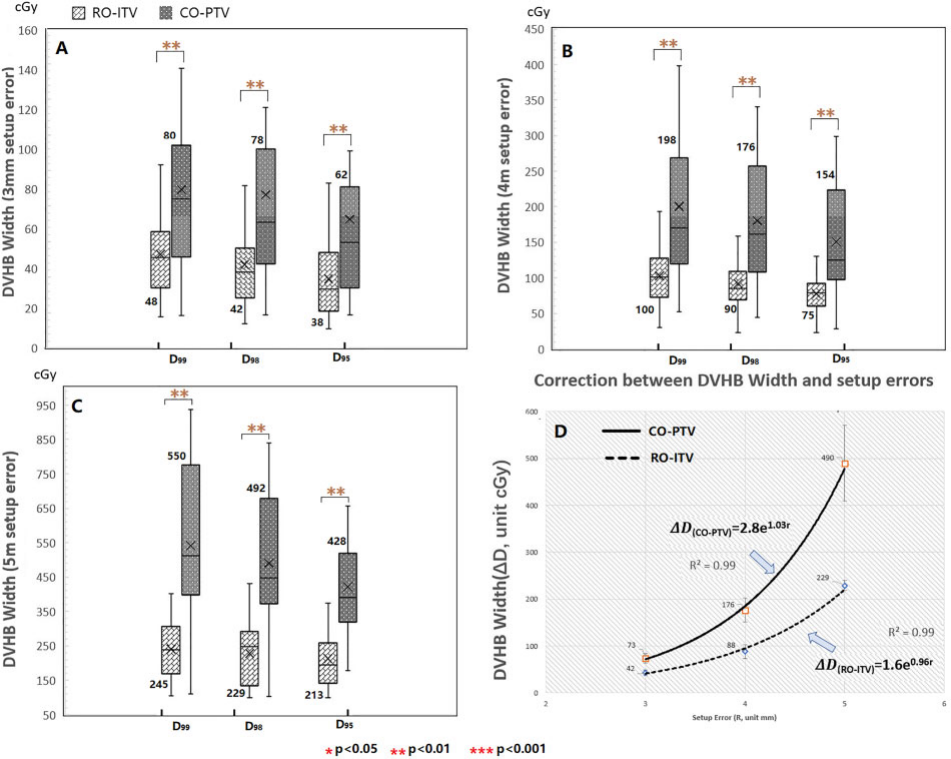
The box plot depicting the statistical result of DVHBW (ΔD) for ITV in *D*_99_, *D*_98_, and *D*_95_ between RO-ITV plan and CO-PTV plan under 3 setup uncertainties group. The center line inside the box is the median value and the cross line represents average value. Correlation analysis between mean DVHBW and setup uncertainties for CO-PTV plan (solid line) and RO-ITV plan (dot line). Results showed probability correlation between the average DVHBW and setup group. CO-PTV indicates planning target volume-based conventional optimization; DVHBW, dose–volume histogram band width; RO-ITV, internal target volume-based robust optimization.

A 5-mm setup uncertainty may be an overestimate due to the updating of immobilization and image-guided devices during patient treatment delivery. Therefore, the effects of 3- and 4-mm setup uncertainties on the plan are also evaluated. Figure 4A-C shows a comparison of the ITV-DVHBW using the DVH metric (*D*_95_, *D*_98_, *D*_99_) under 3-, 4-, and 5- setup groups between the RO-ITV and CO-PTV plans and with average values for all directions. Further details include:

1. For the 3-mm setup group, it was observed that the mean DVHBW is smaller in RO-ITV: for ITV (*D*_99_), it was 48 cGy for RO-ITV versus 80 cGy for CO-PTV (*P* < .01); for ITV (*D*_98_), it was 42 cGy for RO-ITV versus 78 cGy for CO-PTV (*P* < .01); and for ITV (*D*_95_), it was 38 cGy for RO-ITV versus 62 cGy for CO-PTV (*P* < .01). On the other hand, the mean DVHBs were reduced from ITV (*D*_99_) to ITV (*D*_95_), due to the sensitivity of *D*_99_ to the setup uncertainty compared to *D*_95_.
2. A narrow DVHBW for RO-ITV and a lower DVHB value for *D*_95_ were also observed for the 4-mm setup and 5-mm setup groups (see Figure 4B and C for specific average values). We also found that the average DVHBW in *D*_95_, *D*_98_, and *D*_99_ increased from the 3-mm setup to the 5-mm setup group (Figure 4D). The average DVHBW was 42 cGy for the RO-ITV plans compared to 73 cGy for the CO-PTV plans in the 3-mm setup group; 88 cGy for the RO-ITV plans compared to 176 cGy for the CO-PTV plans in the 4-mm setup group; 229 cGy for the RO-ITV plans to 490 cGy for the CO-PTV plans in the 5-mm setup group.
3. A statistical correlation study was performed to evaluate the correlation between the average DVHBW and the setup group and showed the existence of an exponential relationship between the 2 (Figure 4D). For RO-ITV, the expression was $\Delta D_{\text{RO-ITV}}=2.8e^{1.03r}$; while for the CO-PTV plans, it was $\Delta D_{\text{CO-PTV}}=1.6e^{0.9r}$, where r  is equal to 3, 4, and 5 mm. It was observed that the DVHBW in CO-PTV plans was much steeper than in RO-ITV plans, which indicated that while SBRT planning against setup uncertainties can be improved by robust optimization methods, this improvement decreases exponentially with increased setup uncertainty.

### Voxel-Wise Dose Distribution

**Figure 5.**
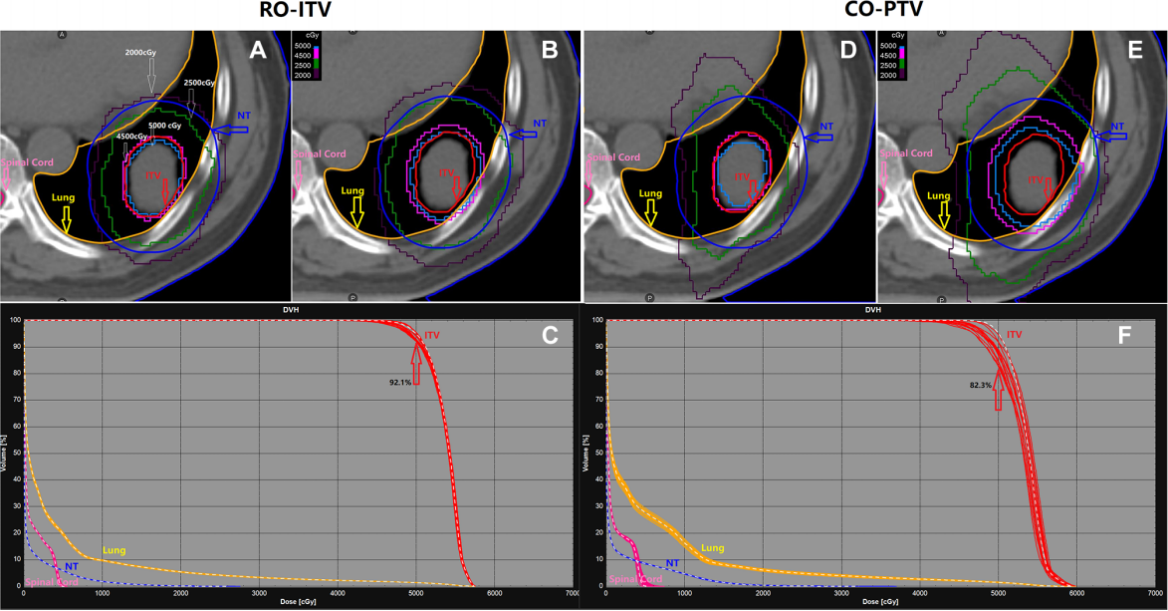
Dose distributions in the transverse plane for a representative patient illustrate the insensitivity of RO-ITV plan (left panels) to set up uncertainties compared with the CO-PTV plan (right panels). The 5000 and 4500 cGy dose line covered a little more ITV volume in RO-ITV plans from Voxel-wise minimum dose distribution (A and D). It was observed that less volume was irradiated in RO-ITV plans according to 2500 and 2000 cGy on Voxel-wise maximum dose distribution (B and E) for NT organ. In addition, RO-ITV plans provided better target coverage in ITV-DVH metric (*V*_100%RX_) than the CO-PTV plans in the worst scenario, as shown in C and F. CO-PTV indicates planning target volume-based conventional optimization; ITV, internal target volume; NT, normal tissue; RO-ITV, internal target volume-based robust optimization.

The VWDD-min/max are used in the transverse plane for a representative patient to illustrate the robustness of RO-ITV plans (Figure 5, right panels) against setup uncertainty when compared with CO-PTV plans (Figure 5, left panels). The 5000 and 4500 dose line covered a greater ITV volume in the RO-ITV plans, as determined by the VWDD-min (Figure 5A and D). It was also observed that less volume was irradiated in the RO-ITV plans using the 2500 cGy dose line on the VWDD-max (Figure 5B and E) for NT organ. In addition, RO-ITV plans provided better target coverage according to the ITV-DVH metric (*V*_100%RX_) than the CO-PTV plans in the worst-case scenario (92.1% vs 82.3%) and are shown in Figure 5C and F.

## Discussion

The purpose of this study is to investigate the feasibility of SBRT planning using RO-ITV methods. To this end, we performed a comprehensive comparison between the RO-ITV and CO-PTV optimization methods based on plan quality and robustness for patients with early stage NSCLC. The goal of lungs SBRT is to deliver an ablative dose to a lung tumor while sparing as much of the surrounding NT as possible. According to the RTOG protocol, the falloff gradient beyond the target extending into NT structure must be steep in all directions.^11^ In the present study, the RO-ITV demonstrated its great potential in maintaining target coverage, while reducing that of NT. The conformality of tumor coverage and intermediate-dose spillage was evidently lower in the RO-ITV plans, as shown in Figure 1. Furthermore, we found that the RO-ITV method performed well in regions where the dose should have been minimal, such as the NT in Figure 2.

Studies have shown that when the biological effective dose is above 100 Gy, the local control and survival significantly improve for patients with early stage NSCLC.^1^ However, RP is a major form of toxicity that limits the increases in the fraction dose in SBRT for lung cancer. Numerous studies have reported that up to 29% SBRT-induced RP cases require clinical intervention.^20,21^ Therefore, such high standards for SBRT have led to much tighter tolerances when treatment planning was performed, and much effort was expended to limit toxicity. Our results show that SBRT plans, using RO-ITV, achieve significant sparing of lung dose compared with the CO-PTV plans, as illustrated by the lung (*D*_mean_, *V*_20_) in Figure 2. The probability of RP was also proved to be lower for RO-ITV (Figure 3). A qualitative explanation of the results goes as follows: typically, the priority in treatment planning is to make sure that the ITV receives the prescribed dose despite uncertainty. In specific cases, especially when the OARs are very close to target and NTs, it is also important for target coverage. Stereotactic body radiotherapy for lung tumors is an example, where sparing of lung NT is relative important to target coverage. In this case, the ITV is expanded by a margin, with a probability distribution that considers setup uncertainty, which might be superior than the plan where ITV is expanded by a fixed margin. Therefore, ITV robust treatment planning creates a plan that easily meets the average dose to the lung.

For the treatment centers which update image-guided devices during treatment delivery, handling the 5-mm setup uncertainty according to the ICRU protocol may result in dose overestimates. It is therefore necessary to consider plan robustness under 3- and 4-mm setup uncertainties. In our studies, 3 groups of setup uncertainties were created to investigate plan robustness, while the proposed DVHBW method was used to provide a simple metric to cross-compare the robustness of SBRT plans based on the RO-ITV and CO-PTV methods. We selected the *D*_99_, *D*_98_, and *D*_95_ ITV as indicators due to their sensitivity to the setup uncertainties. The ITV (*D*_99_) is more sensitive than the ITV (*D*_95_) due to the need of more voxels to account for setup uncertainties, while ITV ( *D*_98_) served as a reference value. Three values of ITV target coverage would be able to reflect the robustness of the plan under setup uncertainties. From the result shown in Figure 4A-C, the ITV-based optimization made the plan more robust against setup uncertainties. This can be explained by the algorithm used in robust optimization, which allows the planner to consider setup uncertainties before performing plan optimization. The robust optimization algorithm discretize setup uncertainties into multiple scenarios. The uncertainty in each scenario is taken into account by the objective function during plan optimization, such that the prescription holds true for each of the various setup uncertainties. An approximative exponential relationship exists between the mean DVHBW and the setup uncertainties, as illustrated in Figure 4D. This relationship indicates that, no matter which optimization method is used, the target coverage will sharply deteriorate with an increased setup uncertainty but the deterioration is slower using RO-ITV plans.

In order to simplify the method of comparing plan robustness of the RO-ITV and CO-PTV plans, DVHBW is used in the target coverage. The approach is different from a root mean square dose (RMSD)-volume histograms (RVH) method,^22,23^ which calculates the RMSD of the 15 doses (ie, 3-mm setup group: nominal scenario [no setup uncertainty] and 14 uncertainty scenarios [6 with 3 mm in X, Y, and Z directions, respectively; and 8 with 3 mm in diagonal directions]) for each voxel within a given structure. The area under the RVH curve in a structure gave a numerical index summarizing plan robustness similar to the way in which the equivalent uniform dose is summarized by a DVH. Thus, plan robustness could also be evaluated by comparing the area under the RVH for the ITV of the RO-ITV and CO-PTV plans. However, RVH is an approach that actually takes into account all the voxels within the ITV; whereas, certain voxels within ITV, for example, prescription isodose surface coverage, are more important in SBRT planning. Voxel wise min/max distribution, as a supplementary method to the DVHB metric, takes into account all the voxels within the patient’s body, from which perturbations in the dose caused by setup uncertainties are distributed at the boundary of the target area (Figure 5).

**Figure 6.**
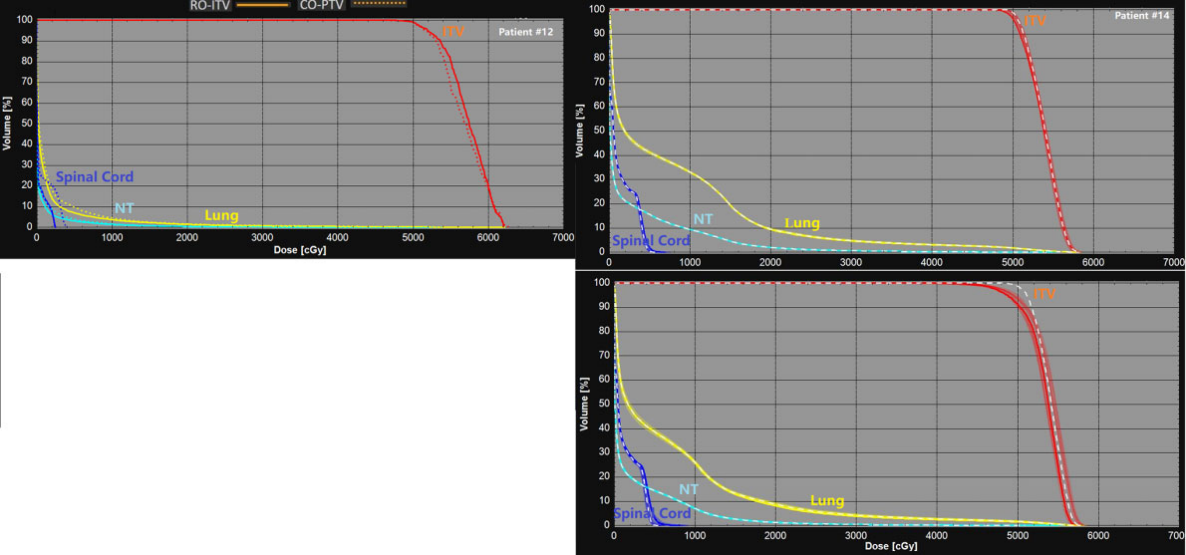
Plan quality comparison (patient no. 12) and plan robustness comparison (patient no. 14) with normalization the plan to 99% coverage of ITV. ITV indicates internal target volume.

Our results provide compelling evidence for the RO-ITV plans, in which plan quality and robustness are obviously advantageous compared to the CO-PTV methods; however, some limitations are worth noting. We assumed that the CO-PTV plan was optimized to PTV coverage of the prescribed dose and then normalized to ITV 95% coverage. This may cause a down normalization of the final CO-PTV with the cost function not being the best design for it. For robust optimized plans, with normalized to 95% coverage of ITV, the cost function may a better fit. Therefore, the compromise method is to normalize the plan to ITV 99% coverage. Due to this normalization that makes RO-ITV plan up and CO-PTV plan slightly down, we picked up 2 extreme cases from the patient’s cohort to verify the conclusion. Figure 6 shows the results in both plan quality (patient no. 12) and robustness (patient no. 14) with the dosimetric details shown in Table 3. Our results show that the conclusion is still valid when normalizing the plan to ITV 99% coverage for the CO-PTV and RO-ITV plans.

**Table 3.** Plan Quality and Robustness Comparison for 2 Representative Patients.

	Patient No. 12		Patient No. 14	
	RO-ITV	CO-PTV	RO-ITV	CO-PTV
CI	1.08	1.12	1.34	1.47
HI	0.18	0.17	0.31	0.26
DFP	143	157	288	369
Lung (*D*_mean_, cGy)	325	340	684	787
Lung (*V*_20_, %)	2.17	2.26	8.73	9.65
Spinal cord (*D*_1cc_, cGy)	203	398	560	628
NT	0.3	0.4	1.46	1.98
NTCP, %	3.1	3.2	4.83	5.46
DVHBW, cGy	192	302	166	329

Abbreviations: CI, conformity index; CO-PTV, planning target volume-based conventional optimization; DFP, dose falloff product; DVHBW, dose–volume histogram band width; HI, homogeneity index; NT, normal tissue; NTCP, normal tissue complication probability.

Another limit is associated with the intrafractional motion issue in treatment of NSCLC-SBRT, which may cause considerable changes in the relative position of the tumor geometry and the MLC aperture, which subsequently has an impact on the dose distribution of the target and the risk to surrounding organs. This is the interplay effect, which had been reported to degrade the quality of the dose delivery.^24,25^ Notably, we did not attempt to consider issues associated with intrafractional motion, although this might be desirable since a similar issue is present in the GTV-to-ITV margin for the ITV-based plan. One group has proposed the use of the difference between the 4D accumulated dose (4DD) and the 4D dynamic accumulated dose (4DDD) to evaluate interplay effects on the treatment plan.^26,27^ The 4DD is a static dose that averages the sum of the doses calculated during the 10 individual phases of a 4DCT image without considering time dependence in the treatment delivery. The 4DDD is a dynamic dose that considers time dependence in the treatment delivery, together with changes in anatomy, owing to respiratory motion. Studies have shown that the 4DDD converges to the 4DD, indicating that its random nature allows the effect of interplay to be averaged out after multiple fractions.^28^ Many studies^29,30^ have also shown that the interplay effect does not significantly affect the cumulative dose distribution over multiple fractions. Besides interplay effects, the erratic motion of target on the treatment day that is not captured on the CT simulation is also not considered here, for example, when a patient coughs or breathes deeply on the linac but not on the CT simulation, the error could be larger in RO-ITV based approach.

## 5. Conclusions

Robust optimization plans provided a better quality plan for target coverage and OAR and for a lower RP according to the lung Lyman-Kutcher-Burman NTCP model. The plan robustness is also shown to be better under different kinds of setup uncertainty scenarios. A novel strategy is introduced to quantitatively evaluate robustness plan using the DVHBW. Our studies show that DVHBW in RO-ITV is significantly lower, which makes the SBRT plan more robust against setup uncertainties. Furthermore, while target coverage sharply deteriorates (approximately exponentially) with increased setup uncertainty, the coverage with RO-ITV is likely to be better than that with CO-ITV.

## Abbreviations

AP: anterior–posterior
CI: conformity index
CO-PTV: PTV-based conventional optimization
CT: computed tomography
CTV: clinical target volume
DFP: dose falloff product
*D*_mean_: mean dose
DVH: dose–volume histogram
DVHBW: dose–volume histogram band width
GTV: Gross target volume
HI: homogeneity index
ICRU: International Commission Radiological Units
IGRT: image-guided radiation therapy
IMRT: intensity modulated radiotherapy
ITV: internal target volume
LR: left–right
MLC: multileaf collimation
NSCLC: non-small cell lung cancer
NT: normal tissue
NTCP: normal tissue complication probability
OAR: organ at risk
PTV: planning target volume
RMSD: root mean square dose
RO-ITV: ITV-based robust optimization
RP: radiation pneumonitis
RTOG: Radiation Therapy Oncology Group
RVH: RMSD volume histogram
SBRT: stereotactic body radiation therapy
t4Dplan: 4-dimensional planning method
VWDD: voxel-wise dose distribution
4-D: 4-dimensional
4DCT: 4-dimensional computed tomography
4DD: 4D accumulated dose
4DDD: 4D dynamic accumulated dose
